# Development of High Tryptophan Maize Near Isogenic Lines Adapted to Temperate Regions through Marker Assisted Selection - Impediments and Benefits

**DOI:** 10.1371/journal.pone.0167635

**Published:** 2016-12-09

**Authors:** Marija Kostadinovic, Dragana Ignjatovic-Micic, Jelena Vancetovic, Danijela Ristic, Sofija Bozinovic, Goran Stankovic, Snezana Mladenovic Drinic

**Affiliations:** 1 Laboratory for Molecular Genetics and Physiology, Maize Research Institute Zemun Polje, Belgrade, Serbia; 2 Group for Breeding of Early Maturity Maize Hybrids with Desirable Qualitative Traits and Male Sterility, Maize Research Institute Zemun Polje, Belgrade, Serbia; 3 Late Maize Hybrid Breeding Group, Maize Research Institute Zemun Polje, Belgrade, Serbia; Julius Kühn-Institut GERMANY

## Abstract

Breeding program aimed at converting standard maize inbred lines to their quality protein maize (QPM) counterparts for growing in temperate climate is being conducted at Maize Research Institute (MRI). The objective of the research presented herein was to develop QPM versions of two commercial ZP inbreds through marker assisted selection (MAS) with *opaque2* specific molecular markers, while maintaining their good agronomic performances and combining abilities. Donor line was a tropical QPM line CML 144. After two backcross and three selfing generations, six near isogenic lines (NILs) with 93% recovery of the recurrent parent genome were created from one cross. Average increments of 30% in tryptophan content and 36% in quality index were obtained, as well as kernels with less than 25% opaque endosperm. Grain yield was increased by 11–31% and combining abilities of the improved lines were on a par with the original line. Correlations between biochemical and agronomic parameters revealed that selection for plant height, ear length and kernel row number together with tryptophan content could be recommended for development of QPM with this material. However, several impediments emerged during selection. Major drawbacks in NIL development were small number of *opaque2* recessive homozygotes (4.5% and 7.6% in BC_2_F_2_ of two crosses) and poor seed set throughout selection, which led to the loss of one cross. Moreover, in the other cross many plants in different generations had to be omitted from further selection due to the insufficient number of kernels. This phenomenon could be explained by incompatibility between pollen and style, possibly due to the exotic donor germplasm. Overall, it could be expected that the use of NILs, which are adapted to temperate climate and have high percentage of domestic germplasm, would outbalance the noted impediments and increase MAS efficiency in different breeding programs.

## Introduction

Maize (*Zea mays* L.) is one of the world’s most important protein sources consumed by humans and animals. However, it is nutritionally imbalanced as the most abundant class of storage proteins (zeins) lack essential amino acids such as lysine, tryptophan and methionine [1]. Most attempts to improve the nutritional quality of maize proteins involve altering zein content, i.e. increasing the ratio of non-zein to zein proteins.

Important researches on maize protein quality improvement took place in the 1960s, after discovery of several mutations (*opaque2—o2*, floury2*—fl2*, *opaque7—o7*, *opaque6—o6* and *floury3—fl3*) that caused a decrease in zein content, which was accompanied by increase of other protein classes as well as by increase in the relative amount of essential amino acids [2–5]. Recessive mutation *o2* has been the most widely studied and used as a source for genetic improvement of the nutritional value of maize proteins. Recessive homozygous genotypes (*o2o2*) have substantially higher lysin and tryptophan content compared to heterozygotes (*O2o2*) or dominant homozygotes (*O2O2*) [6]. However, pleiotropic effect of the *o2* mutation makes the maize endosperm soft and susceptible to cracking, ear rots and storage pests. The genes controlling the soft and starchy texture of *o2* endosperm are designated as *opaque2* modifiers (*Opm*) and they have been proved to be genetically complex in nature [7]. Breeding for high quality protein maize requires yet another, distinctive genetic system comprised of minor modifying loci and these modifiers/enhancers confer higher lysine/tryptophan content in maize [8]. Interdisciplinary research team from the International Maize and Wheat Improvement Center (CIMMYT), Mexico, created through conventional breeding programs the new, agronomically acceptable and nutritionally improved *opaque2* types named Quality Protein Maize—QPM [9].

QPM was primarily developed for the regions where maize is staple food and where availability of other protein sources is scarce [10]. Besides higher protein quality, QPM also has other nutritional advantages over standard maize [11–13] and thus can significantly improve nutritional status of sensitive groups. On the other hand, QPM is used as animal feed in countries in which meat consumption per capita is high. It was presented in many studies that QPM had a positive overall impact on the weight gain of both poultry and pigs [14–16]. QPM could also substitute soybean meals and synthetic lysine in feed composites for poultry and pigs, resulting in considerable savings in feed production [17, 18].

Although QPM was created through conventional breeding, marker assisted selection (MAS) has been increasingly used for improvement of protein quality in maize. Phi057, phi112 and umc1066 SSR (simple sequence repeats) markers, located within the *opaque2* gene, are used to distinguish between recessive and dominant alleles [19]. Foreground selection with these markers enables maintenance of recessive genes without the need for progeny testing in each generation of selection, as homozygous and heterozygous plants can be distinguished using specific SSR markers. During backcrossing, DNA markers can help in reducing the number of generations required to recover a recurrent parent’s genome. There are several successful examples of MAS in QPM breeding, mostly for growing in tropical and sub-tropical regions. Thus, in Babu et al. [20] it was presented that the development of QPM lines can be obtained by two-generation backcrossing followed by two generations of selfing. *Opaque2* targeted foreground selection, as well as background selection, was performed in adequate backcross generations, while phenotypic selection for endosperm hardness, tryptophan content and desirable agronomic traits were performed in selfing generations.

The results presented in this paper are a part of the breeding program aimed at increasing protein quality of maize inbred lines and hybrids, which is being conducted at the Maize Research Institute (MRI) Zemun Polje [21–25]. The main objective of the research presented herein was to develop high quality protein maize lines adapted to temperate regions from commercial ZP inbred lines using *opaque2* specific molecular markers, while maintaining their good agronomic performances and combining abilities.

## Materials and Methods

### Plant material

Two MRI commercial inbred lines (ZPL 3 and ZPL 5) were chosen as recurrent parents. ZPL 3 and ZPL 5 are orange, dent-like lines adapted to the local environmental conditions in Serbia. Due to their excellent combining abilities, they are components of the leading MRI hybrids. Donor line was CML 144, one of the CIMMYT’s most successful tropical QPM lines. CML 144 is white flint with excellent combining ability.

The choice of parental lines was based on the presence/absence of *o2* allele and difference in tryptophan content between the QPM and standard maize inbred lines. As presented in Kostadinovic et al. [25], tryptophan content was almost two times higher in CML 144 (0.093%) than in ZPL 3(0.052%) or ZPL 5 (0.054%), while phi057 and umc1066 were able to distinguish ZPL 3 and ZPL 5 (*O2O2)* from CML144 (*o2o2*) and were used for foreground selection throughout the MAS.

### Outline of the conversion of standard maize line to its QPM version

**Fig 1:**
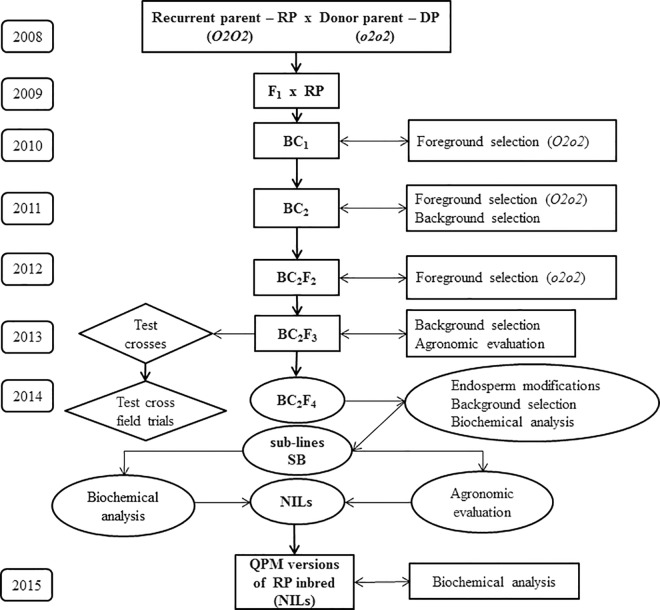
Schematic presentation of MAS for conversion of standard maize inbred lines to their QPM counterparts. On the left–year of each experiment and trial performance. ↓ arrow indicates backcross or selfing; ↔ arrow indicates that the results of the analysis were used for selecting plants for next generation. NILs–Near Isogenic Lines.

The conversion process (Fig 1) included two generations of backcrossing and three generations of selfing. The experiment was conducted in Zemun Polje, Serbia, from 2009 to 2015.

F_1_ plants (ZPL 3 × CML 144 and ZPL 5 × CML 144) were backcrossed with recurrent parent lines to generate BC_1_ progeny in 2009. The following year, genotypes that were heterozygous for the gene-specific phi057 and umc1066 loci were backcrossed with the recurrent parents to produce BC_2_ progeny.

A two-level selection procedure was carried out in BC_2_ during 2011. First, BC_2_ plants were screened at phi057 and umc1066 loci to identify *O2o2* heterozygotes. Second, the selected heterozygotes were screened with SSR markers distributed across all 10 chromosomes. Genotypes with the highest recovery of recurrent parents’ genome were selected among the heterozygotes and selfed to produce kernels of BC_2_F_2_ progenies.

In 2012, DNA samples from BC_2_F_2_ progenies were collected and subjected to foreground selection before flowering to identify plants that attained recessive homozygosity at *o2* locus. The genotypes having homozygous recessive *opaque2* locus were selfed to produce BC_2_F_3_ families.

BC_2_F_3_ families were subjected to the whole genome background selection and at the same time their agronomical evaluation was done in a field trial alongside the recurrent parent in 2013. Plants within selected families were used the same year as fathers for crosses with commercial ZP tester in order to compare their combining abilities with the original standard line. They were also selfed and kernels of BC_2_F_4_ families were produced. BC_2_F_3_ families with highest genetic and phenotypic similarities with the recurrent parent were chosen for further selection. Kernels of successfully selfed ears (i.e. BC_2_F_4_ families) were separately kept for further analysis.

The selfed ears (i.e. BC_2_F_4_ families) were subjected to endosperm modification, SSR and biochemical analysis. Based on the results of these analyses, the best BC_2_F_4_ families were chosen for a one-year field trial conducted in 2014, aimed at comparing their agronomic performances with the recurrent parent and were designated as sub-lines (SLs). Also, test crosses were sawn in the same year for the agronomic evaluation. Furthermore, plants of the sub-lines were selfed, and based on their agronomic and biochemical performances 13 progenies were selected and assigned as Near Isogenic Lines (NILs). NILs were sawn for the multiplication in 2015, together with original ZPL 5 line. Seven NILs failed to produce seed, while remaining six represented high-tryptophan versions of the ZPL 5 line with good agronomic performances.

### Molecular analysis

#### DNA isolation

Genomic DNA was isolated according to Doyle and Doyle [26]. Samples were prepared from the leaves of individual plants (BC_1_, BC_2_ and BC_2_F_2_) and by pooling 20 leaves (BC_2_F_3_) or 20 kernels (BC_2_F_4_ and NILs) per sample. DNA was quantified using spectrophotometer (UV-1601, Shimadzu) and samples were stored at -20°C until use.

#### Foreground selection using opaque2 specific markers

Identification of heterozygous and homozygous plants was done with phi057 and umc1066 specific SSR markers. PCR reaction, fragment amplification, electrophoresis and documentation were carried out as presented in Kostadinovic et al [25]. The amplification products were determined based on the positions of the bands relative to the standard (dominant allele) and QPM (recessive allele) lines used as controls.

#### Background selection using SSR markers

SSR analysis was performed with 50 primer pairs (S1 Table) evenly distributed throughout the maize genome, selected from the maize database (www.maizegdb.org). PCR reaction was prepared as given in Kostadinovic et al [25]. Amplification was performed in thermocycler Biometra TProfessional Standard 96 with the following touch-down program: an initial denaturation at 95°C/5 min, followed by 15 cycles each of denaturation at 95°C/30 s, annealing at 63.5°C/1 min (-0.5°C/cycle) and extension at 72°C/1 min; another 22 cycles of 95°C/30 s, 56°C/1 min and 72°C/1 min were performed. Final elongation was at 72°C for 4 min. Electrophoresis and documentation were performed as in foreground selection, with 20bp DNA ladder as a marker. The amplified fragments were binary scored.

### Biochemical analysis

#### Sample preparation

Samples for protein and tryptophan content analysis were prepared as shown in Kostadinovic et al [25]. Shortly, two sub-samples (30 kernels each) per genotype were dried at 65°C over night, milled and flour was defatted by hexane treatment in Soxhlet extractor.

#### Total protein and tryptophan content

Protein content was determined by the standard Kjedahl method which is based on nitrogen determination as explained in Vivek et al. [8], while tryptophan content was determined by the colorimetric method given in Nurit et al. [27]. Based on the results of protein and tryptophan content determination, quality index (QI), defined as tryptophan to protein ratio in the sample, was calculated as QI = 100 × (tryptophan content in the sample / protein content in the sample).

### Agronomic and phenotypic analysis

#### Endosperm modifications

Kernel endosperm modifications were visually assessed using light table, according to the scoring scale from 1 (completely translucent, with no opaqueness) to 5 (completely opaque), as presented in Vivek et al. [8]. Modification score 2 was given to the kernels that were 25% opaque, while scores 3 and 4 were given to 50% and 75% opaque kernels, respectively. Kernels with scores 2 and 3 were selected in BC_1_ and BC_2_ generations, while kernels scored 1 and 2 were selected in the advanced generations.

#### Field trials

Field trials were conducted for estimation of agronomic performances of BC_2_F_3_ families in comparison with original ZPL 5 line in 2013 and BC_2_F_4_ families (i.e. sub-lines) in 2014. Separate trials with the same families were conducted alongside the main trials for further selfing and background selection. Each successfully selfed ear from these trials was shelled and kernels maintained separately for biochemical analysis and production of the next generation. Test-crosses of the selected BC_2_F_3_ progenies were tested for combining ability with the check commercial hybrid representing ZPL 5 line test cross, in 2014.

All field trials were set up according to the randomized complete block (RCBD) design in two replications at two locations nearby Zemun Polje, Belgrade, Serbia. No specific permissions was required for any locations/activities and the field studies did not involve endangered or protected species. Plant densities were 80,000 plants ha^-1^ for the trials with selected families and 67,000 plants ha^-1^ for the test crosses. Genotypes in the main trials were sown in one-row plots consisting of 20 plants each, while in the trials for family selfing the plots consisted of 10 plants. Standard agronomic practices were used to provide adequate nutrition and keep the fields free of weeds and diseases.

A total of 15 traits were scored: plant height (PH), ear height (EH), leaf number (LN), leaf number above the uppermost ear (LNE), ear number per plant (ENP), percentage of broken plants (BP), anthesis-silking interval (ASI), ear length (EL), kernel row number (KRN), number of kernels per row (NKR), kernel length (KL), hundred kernel weight (HKW), cob percentage (CP), grain moisture (GM), and grain yield (GY). Ten competitive plants per plot were randomly chosen for the traits estimation, except for BP and GY for which all plants per plot were used. Immediately prior to harvest, plots were counted for stalk lodging (percentage of plants broken below the uppermost ear). At physiological maturity, plots were hand-harvested. Average moisture content was measured and this data was used to calculate grain yield in t ha^-1^ adjusted to 14% moisture.

### Statistical analysis

#### Genetic distance

SSR profiles were scored as presence/absence of fragments in each sample and the data was assembled into a binary matrix. Genetic similarity (GS) was calculated in accordance with Dice [28] using statistical NTSYSpc2 program package [29].

#### Biochemical analysis and field trials

For biochemical traits, as well as for agronomic traits from the trials conducted in one year each, one factorial analysis of variance (ANOVA) according to RCBD was performed. Fisher’s least significant difference test (LSD) was used to estimate the significances of differences between the observed means. All statistical analyses were done in MSTAT-C software [30]. Pearson’s correlation coefficients were calculated between the estimated traits.

## Results

### Foreground and background selection in the backcross generations

One hundred BC_1_ plants were obtained from the cross ZPL 3 × CML 144. PCR amplification with phi057 and umc1066 specific *opaque2* markers identified 52 heterozygous individuals (52%) that were used for backcrossing with ZPL 3 and developing BC_2_ generation. In BC_2_, the specific markers identified 109 out of 227 plants (48%) as heterozygous. In the cross ZPL 5 × CML 144, 47 out of 100 BC_1_ plants (47%) and in BC_2_ 29 out of 67 plants (43.3%) were heterozygous.

Background selection was performed on BC_2_ heterozygous plants. All genetic similarities were calculated using Dice coefficient. Genetic similarity values between ZPL 3 and its BC_2_ plants were in the range from 0.51 to 0.89, with the average value of 0.74. Genetic similarities between ZPL 5 and its BC_2_ plants ranged from 0.67 to 0.85, the average value being 0.75. A total of five BC_2_F_2_ progenies (selfed BC_2_ plants) with the highest GS values were chosen for further work—three progenies derived from ZPL 3 × CML 144 cross (with GS values of 0.89, 0.87 and 0.86) and two from ZPL 5 × CML 144 cross (with GS values 0.85 and 0.80).

### SSR, phenotypic and biochemical analysis of selfed generations

Although 15 recessive o*2o2* homozygotes out of 329 plants (4.5%) were identified in CML 144 × ZPL 3 BC_2_F_2_ progenies, none of them produced sufficient number of kernels to develop next generation. For this reason, selection with ZPL 3 × CML 144 had to be abandoned.

In BC_2_F_2_ progenies of ZPL 5 × CML 144, 19 recessive o*2o2* homozygotes out of 250 plants (7.6%) were obtained. MAS was continued with ten homozygous plants that had sufficient kernel number after selfing (comprising BC_2_F_3_ families, further designated with F).

The ten BC_2_F_3_ families were submitted to both phenotypic and SSR analysis. No significant differences were revealed between parental line ZPL 5 and the analysed families in the field trial in 2013 for the following traits: ASI, LNE, ENP, BP, CP, NKR and HKW (data not shown). On the other hand, significant differences (p<0.05) were found between ZPL 5 and all the families for PH, LN and KRN. The highest number of traits (12 out of 15) not significantly different from ZPL 5 was found in families F-5 and F-6.

SSR analysis showed that genetic similarity values between the recurrent parent ZPL 5 and the ten BC_2_F_3_ families ranged from 0.71 to 0.94, with the average value of 0.81. The highest proportion of the recurrent parent's genome was found in F-5 (0.94) and F-6 (0.91), followed by F-4 (0.84), and F-1 (0.82). These four families were chosen for further work.

A total of 11 BC_2_F_4_ families were obtained with sufficient number of kernels for endosperm modifications, biochemical and SSR analysis (Table 1). Six progenies (F-1/1, F-4/1, F-5/1, F-5/2, F-5/3 and F-5/4) had exclusively hard endosperm kernels (≤ 25% opaque). Percentage of soft endosperm kernels within the other five progenies was in the range from 8.8% (F-1/2) to 16.5% (F-6/4). Completely opaque endosperm kernels were identified in a small percentage, from 2.2% (F-6/4) to 3.8% (F-6/3). Biochemical analysis of BC_2_F_4_ families showed significant increase (p<0.05) of tryptophan content and quality index relative to the recurrent parent. Tryptophan content ranged from 0.072 (F-4/1 and F-5/3) to 0.086 (F-1/2) and QI from 0.61 (F-4/1) to 0.76 (F-1/2).

**Table 1:** Endosperm Modifications, Biochemical Characterisation and Genetic Similarities of BC_2_F_4_ Families.

Generation		SL^a^	Endosperm modification				Biochemical traits			GS^e^
BC_2_F_3_	BC_2_F_4_		≤ 25%	≤ 50%	≤ 75%	100%	TC^b^ (%)	PT^c^ (%)	QI^d^	
ZPL 5	-	-	100	-	-	-	0.060f	11.56ab	0.52h	1
F-1	F-1/1	-	100	-	-	-	0.073cde	11.49ab	0.64cdefg	0.81
	F-1/2	-	91.2	4.4	2.0	2.4	0.086a	11.26abc	0.76a	0.81
**F-4**	**F-4/1**	**SL-4/1**	**100**	**-**	**-**	**-**	**0.072cde**	**11.74a**	**0.61fg**	**0.91**
**F-5**	**F-5/1**	**SL-5/1**	**100**	**-**	**-**	**-**	**0.073bcde**	**10.62cd**	**0.69bcde**	**0.92**
	**F-5/2**	**SL-5/2**	**100**	**-**	**-**	**-**	**0.075bcd**	**11.31ab**	**0.66bcdef**	**0.91**
	**F-5/3**	**SL-5/3**	**100**	**-**	**-**	**-**	**0.072cde**	**11.02bcd**	**0.66bcdef**	**0.91**
	**F-5/4**	**SL-5/4**	**100**	**-**	**-**	**-**	**0.073bcde**	**10.51d**	**0.70abc**	**0.92**
**F-6**	F-6/1	-	91	6.3	2.7	-	0.073cde	11.61ab	0.63efg	0.91
	**F-6/2**	**SL-6/2**	**88.3**	**8.3**	**3.4**	**-**	**0.073bcde**	**10.51d**	**0.69abcd**	**0.92**
	**F-6/3**	**SL-6/3**	**91.3**	**1.3**	**3.6**	**3.8**	**0.077bc**	**11.82a**	**0.66bcdef**	**0.91**
	**F-6/4**	**SL-6/4**	**83.5**	**11.9**	**2.4**	**2.2**	**0.080ab**	**11.20abc**	**0.71ab**	**0.93**
mean	-	-	-	-	-	-	0.073	11.25	0.65	-
SD^f^	-	-	-	-	-	-	0.01	0.49	0.06	-
CV^g^ (%)	-	-	-	-	-	-	4.60	2.63	4.50	-
LSD_0.05_^h^	-	-	-	-	-	-	0.01	0.64	0.07	-

^a^SL–chosen BC_2_F_4_ families designated as sub-lines

^b^TC—tryptophan content

^c^PT—protein content

^d^QI—quality index

^e^GS—genetic similarity

^f^SD—standard deviation

^g^CV—coefficient of variation

^h^LSD_0.05_—least significant difference at 0.05 level.

Means followed by the same letter(s) within the same columns are not significantly different at 0.05 level. Bolded are progenies used for further selection.

Genetic similarity values between the recurrent parent and the 11 BC_2_F_4_ families ranged from 0.81 to 0.93 (Table 1), with the average value of 0.90. Nine out of 11 progenies with GS values higher than the average were identified. They include: F-6/4 with the highest GS of 0.93, followed by F-5/1, F-5/4 and F-6/2 with GS of 0.92, while F-4/1, F-5/2, F-5/3, F-6/1 and F-6/3 had GS of 0.91.

Nine BC_2_F_4_ families with highest GS, which also had significantly increased tryptophan content and high percentage of hard endosperm, represented the improved versions of standard maize ZPL 5 inbred line and were designated as sub-lines (Table 1). Sub-line SL-6/1 was discarded due to the insufficient kernel quantity and the remaining eight sub-lines were used for further work.

The eight chosen improved sub-lines were submitted to phenotypic, biochemical and SSR analysis in 2014. Phenotypic analysis revealed no significant differences between the parental line and the analyzed sub-lines for the following traits: ASI, BP and CP (S2 Table). For two traits—PH and KRN significant differences (p<0.05) were found between the recurrent parent and all the analyzed sub-lines. Significant differences (p<0.05) between ZPL 5 and some sub-lines were found for LN (SL-4/1, SL-6/2 and SL-6/3), LNE (SL-4/1, SL-5/3, SL-6/2 and SL-6/3), EL (SL-4/1, SL-5/2, SL-5/4, SL-6/3 and SL-6/4), as well as for EH, ENP, GM, NKR, KL, HKW and GY (SL-4/1). The highest number of traits (13 out of 15) not significantly different from ZPL 5 was found for SL-5/1. Grain yield (t ha^-1^) for each sub-line is given in Table 2. It can be seen that the increase in percentage of grain yield, which ranged from 11 to 172% was found in all sub-lines compared to ZPL 5, but it was not significant (p<0.05) for most of them. However, the only exception was regarding SL-4/1, for which a significant increase was noted.

**Table 2:** Biochemical Analysis, Endosperm Modification and Grain Yield of the Improved Sub-Lines.

Genotype	NP^a^	TC^b^ (%)		PC^c^ (%)		QI^d^		EM^e^ (%)	GY^f^ (t ha^-1^)	GYC^g^ (%)
		Average	Range	Average	Range	Average	Range			
ZPL 5		0.060	0.060b	11.56	11.56cd	0.52	0.52q	-	3.16de	-
SL-4/1	7	0.075	0.067ab-0.078ab	11.59	11.39ef-11.97a	0.65	0.58p-0.68hi	100	8.59a	172
SL-5/1	4	0.072	0.070ab-0.074ab	11.19	10.75j-11.46def	0.64	0.62mno-0.66jk	97.84	2.46e	-22
**SL-5/2**	**7**	**0.074**	**0.071ab-0.080ab**	**10.6**	**10.21n-11.09gh**	**0.70**	**0.68hi-0.72bcd**	**100**	**3.77cde**	**19**
**SL-5/3**	**5**	**0.077**	**0.073ab-0.081a**	**10.61**	**10.41m-10.98hi**	**0.73**	**0.70defg-0.75a**	**100**	**3.50cde**	**11**
**SL-5/4**	**7**	**0.075**	**0.071ab-0.077ab**	**10.62**	**10.02o-11.00hi**	**0.71**	**0.69ghi-0.74ab**	**100**	**4.14bcd**	**31**
SL-6/2	1	0.070	0.070ab	10.18	10.18n	0.69	0.69ghi	90.77	4.26bcd	35
SL-6/3	2	0.076	0.068ab-0.085a	10.84	10.54kl-11.14g	0.70	0.65klm-0.76a	77.34	3.50cde	11
**SL-6/4**	**2**	**0.070**	**0.066ab-0.075ab**	**10.24**	**10.11no-10.37m**	**0.68**	**0.65kl-0.72bc**	**85.25**	**3.88cde**	**23**
mean	-	-	0.074	-	10.87	-	0.68	-	4.32	
SD^h^	-	-	0.01	-	0.52	-	0.05	-	1.93	
CV^i^(%)	-	-	2.98	-	0.55	-	2.8	-	23.21	
LSD_0.05_^j^	-	-	0.02	-	0.13	-	0.02	-	1.58	

^a^NP—number of selfed plants per SL

^b^TC—tryptophan content

^c^PC—protein content

^d^QI—quality index

^e^EM—endosperm modification

^f^GY–grain yield

^g^GYC- percentage of grain yield change

^h^SD—standard deviation

^i^CV—coefficient of variation

^j^LSD_0.05_—least significant difference at 0.05 level.

Means followed by the same letter(s) within the same columns are not significantly different at 0.05 level. Bolded are sub-lines within which Near Isogenic Lines (NILs) were identified.

Considering kernel modifications, four out of eight analyzed sub-lines (SL-4/1, SL-5/2, SL-5/3 and SL-5/4) had 100% hard endosperm kernels (Table 2). Biochemical analysis of selfed individual plants within the sub-lines showed significant increase of average tryptophan content and quality index, as well as significant decrease in protein content relative to ZPL 5 at p<0.05. Average tryptophan content was increased from 17% (SL-6/2 and SL-6/4) to 28% (SL-5/3). Likewise, average QI was increased from 23% (SL-5/1) to 40% (SL-5/3). Average protein content was decreased in most sub-lines. Average values and range of biochemical traits for each sub-line are given in Table 2.

After SSR analysis and GS estimation with 50 SSR markers (a total of 63 alleles and average of 1.97 alleles per locus) using Dice coefficient, as well as kernel modification scoring, 13 progenies (deriving from SL-5/2, SL-5/3, SL-5/4 and SL-6/4) were identified that also met tryptophan content threshold for QPM. Genetic similarity of all 13 progenies to ZPL 5 was 0.93. These 13 progenies were referred to as Near Isogenic Lines (NILs) and designated NIL-1 to NIL-13. Tryptophan content was in the range from 0.075 (in four NILs) to 0.81 (in one NIL) and their kernels were exclusively type 1 (less than 25% opaque). NILs were sown together with ZPL 5, selfed and analysed for biochemical characteristics in 2015. Seven of them failed to produce enough kernels and could not be analyzed, leaving a total of six NILs with moderate seed set as the final result of the selection. At the same time, original line had a full seed set. The results of biochemical analysis of NILs in two different years are given in Table 3.

**Table 3:** Tryptophan Content, Protein Content and Quality Index of Near Isogenic Lines (Nils) per year.

SL^a^	Genotype	Year												Average change for both years (%)		
		2014						2015								
		Tryptophan (%)		Protein (%)		Quality Index		Tryptophan (%)		Protein (%)		Quality Index				
		TC^b^	CP^c^	PC^d^	CP	QI^e^	CP	TC	CP	PC	CP	QI	CP	TC	PC	QI
/	ZPL 5	0.060c	/	11.56a	/	0.52g	/	0.057c	/	10.36cd	/	0.55c	/	/	/	/
SL-5/2	NIL-1	0.080ab	33	11.09b	-4	0.72cd	38	/	/	/	/	/	/	/	/	/
SL-5/2	NIL-2	0.074b	23	10.96b	-5	0.68f	31	/	/	/	/	/	/	/	/	/
SL-5/2	**NIL-3**	**0.075ab**	**25**	**10.44d**	**-10**	**0.72cd**	**38**	**0.075b**	**32**	**10.27d**	**-1**	**0.73b**	**33**	**28.2**	**-6**	**36**
SL-5/2	**NIL-4**	**0.077ab**	**28**	**10.65c**	**-8**	**0.72cd**	**38**	**0.074b**	**30**	**10.28d**	**-1**	**0.72b**	**31**	**29**	**-5**	**35**
SL-5/3	NIL-5	0.081a	35	10.98b	-5	0.74abc	42	/	/	/	/	/	/	/	/	/
SL-5/3	NIL-6	0.080a	33	10.73c	-7	0.75a	44	/	/	/	/	/	/	/	/	/
SL-5/3	NIL-7	0.076ab	27	10.47d	-9	0.73bcd	40	/	/	/	/	/	/	/	/	/
SL-5/3	**NIL-8**	**0.076ab**	**27**	**10.97b**	**-5**	**0.69ef**	**33**	**0.075b**	**30**	**10.56b**	**2**	**0.71b**	**29**	**29**	**-2**	**31**
SL-5/4	**NIL-9**	**0.075ab**	**25**	**10.02e**	**-13**	**0.74ab**	**42**	**0.086a**	**51**	**10.90a**	**5**	**0.78a**	**42**	**37.6**	**-5**	**42**
SL-5/4	**NIL-10**	**0.077ab**	**28**	**10.73c**	**-7**	**0.72de**	**38**	**0.074b**	**30**	**10.41bcd**	**0**	**0.73b**	**33**	**29**	**-4**	**36**
SL-5/4	NIL-11	0.076ab	27	11.00b	-5	0.69f	33	/	/	/	/	/	/	/	/	/
SL-5/4	**NIL-12**	**0.075ab**	**25**	**10.39d**	**-10**	**0.71cd**	**37**	**0.077b**	**35**	**10.55bc**	**2**	**0.73b**	**33**	**29.9**	**-4**	**35**
SL-6/4	NIL-13	0.075ab	25	10.37d	-10	0.72bcd	38	/	/	/	/	/	/	/	/	/
mean	/	0.075	/	10.74	/	0.70	/	0.074	/	10.48	/	0.71	/	/	/	/
SD^f^	/	0.01	/	0.39	/	0.06	/	0.01	/	0.21	/	0.07	/	/	/	/
CV^g^(%)	/	2.90	/	0.71	/	3.00	/	2.33	/	0.41	/	2.44	/	/	/	/
LSD_0.05_^h^	/	0.01	/	0.17	/	0.02	/	0.01	/	0.19	/	0.02	/	/	/	/

^a^SL—sub-lines

^b^TC—tryptophan content

^c^CP—Change compared to ZPL 5

^d^PC—protein content

^e^QI—quality index

^f^SD—standard deviation

^g^CV—coefficient of variation

^h^LSD_0.05_—least significant difference at 0.05 level.

Means followed by the same letter(s) within the same columns are not significantly different at 0.05 level, "-" indicates decrease.

### Correlations between phenotypic and biochemical traits of BC_2_F_4_ sub-lines

Statistical significance of Pearson correlation coefficients between biochemical and agronomic traits of the selected sub-lines are shown in Table 4. Correlations between biochemical and majority of phenotypic and agronomic traits were not significant. Highly significant correlations (p<0.01) were found between TC and PH, as well as KRN, and significant correlations (p<0.05) were found between TC and LNE, EL and NKR. Protein content showed highly significant correlations (p<0.01) with GM and HKW and significant correlation (p<0.05) with KRN. Highly significant correlation (p<0.01) was identified between QI and KRN, and significant correlations (p<0.05) were found between QI and PH, as well as QI and EL.

**Table 4:** Statistical Significance of Correlations Between Biochemical and Agronomic Traits of Selected Sub-Lines.

	ASI^a^	PH^b^	EH^c^	LN^d^	LNE^e^	ENP^f^	BP^g^	CP^h^	GM^i^	EL^j^	KRN^k^	NKR^l^	KL^m^	HKW^n^	GY^o^
TC^p^	0.017^ns^	0.513**	0.217^ns^	0.178^ns^	0.367*	0.160^ns^	-0.230^ns^	-0.103^ns^	0.169^ns^	0.409*	0.562**	0.363*	0.187^ns^	0.060^ns^	0.252^ns^
PC^q^	-0.046^ns^	-0.048^ns^	0.310^ns^	0.313^ns^	0.070^ns^	0.055^ns^	-0.230^ns^	0.285^ns^	0.470**	-0.096^ns^	-0.392*	-0.028^ns^	0.251^ns^	0.572**	0.268^ns^
QI^r^	0.026^ns^	0.410*	0.002^ns^	-0.021^ns^	0.249^ns^	0.078^ns^	-0.044^ns^	-0.221^ns^	-0.131^ns^	0.344*	0.620**	0.282^ns^	0.001^ns^	-0.270^ns^	0.046^ns^

^a^ASI—anthesis-silking interval

^b^PH—plant height

^c^EH—ear height

^d^LN—leaf number

^e^LNE—leaf number above the uppermost ear

^f^ENP—ear number per plant

^g^BP—percentage of broken plants

^h^CP—cob percentage

^i^GM—grain moisture

^j^EL—ear length

^k^KRN—kernel row number

^l^NKR—number of kernels per row

^m^KL—kernel length

^n^HKW—hundred kernel weight

^o^GY—grain yield

^p^TC—tryptophan content

^q^PC—protein content

^r^QI—quality index

ns—not significant

* and **—significant at 0.05 and 0.01 level, respectively.

### Combining abilities of the selected BC_2_F_3_ families

Combining abilities of the four selected BC_2_F_3_ families were compared with combining ability of ZPL 5 in crosses with a common commercial ZP tester. The majority of the tested agronomic traits showed no significant differences (S3 Table) between the hybrid created with ZPL 5 (H-1) and hybrids with selected BC_2_F_3_ families F-1, F-4, F-5 and F-6, respectively (hybrids H-2 to H-5). Significant differences (p<0.05) were found for PH (H-2 and H-4), LNE (H-3 and H-4), GM (H-3) and KRN (H-2, H-3, H-4 and H-5). The highest number of traits (14 out of 15) not significantly different from H-1 was found for H-5 (created with F-6).

## Discussion

The main advantages of MAS for developing QPM lines are direct selection of target *o2* gene with specific SSR markers (foreground selection) and fast recovery of recurrent parent's genome (background selection). As reported in Kostadinovic et al. [25], the recipient parental ZPL 3 and ZPL 5 lines were clearly distinguishable with *o2* SSR markers phi057 and umc1066 from the donor line CML 144, and thus were used for foreground selection. The results of foreground and background selection indicated a well-designed and performed experiment. Percentage of heterozygous plants identified in BC_1_ and BC_2_ generations was approximately 50%, which is in accordance with the expected Mendelian ratio of 1 *O2O2*: 1 *O2o2* in backcross generations. A total of 50 SSR markers evenly distributed throughout the genome (at least three per chromosome) were efficiently used for identification of the genotypes with the highest proportion of recurrent parent's genome in BC_2_, BC_2_F_3_ and BC_2_F_4_ generations. In BC_2_ generation, 65% plants had genetic similarity with recurrent parent about the average value. Their number decreased to the upper and lower extremes of genetic similarity coefficient values. According to Babu et al [20], care needs to be taken in a practical MAS program to avoid sampling error and exaggerated estimates of recurrent parent genome associated with smaller number of marker data points. However, almost symmetrical distribution of individuals with low, moderate and high recurrent parent's genome content in the BC_2_ generation indicated unbiased sampling and sufficient number of marker data points. Also, what has to be taken into account when applying MAS in practical breeding is the fact that experiments should be on a case-by-case basis and that it is important to consider the nature of the germplasm involved (e.g. agronomic quality and number of lines to be converted) and the technical options available at the marker level [31]. In our research, two BC_2_F_3_ families with the highest genetic similarities also had the highest phenotypic similarities with the original ZPL 5 line. This justifies using SSR markers in MAS and proves that 50 SSR markers in this research were properly chosen.

Gupta et al. [32] used MAS for development of QPM parental lines of Vivek-9 hybrid and developed QPM hybrid in less than half the time required through conventional breeding. The recovery of the recipient genome in their best lines varied from 83.7% to 94.4% for one and from 80% to 93.7% for another type of cross. Babu et al. [20] obtained three plants with 93% to 96% recurrent parent's genome during conversion of V25 inbred line. However, in both of these researches maximum recovery was obtained in BC_2_, while the recovery of 93% of ZPL 5 genome was accomplished in BC_2_F_4_. These differences in the pace of recurrent parent genome recovery were due to greater genetic similarity between donor and recipient lines used in their researches. In our work, maximal genetic similarity in BC_2_ was 0.85 and it increased in subsequent three selfing generations as a consequence of random genetic recombination.

Marker assisted selection in QPM breeding programs is also aided by two rapid, inexpensive and reliable assays—tryptophan content determination in maize grain and endosperm modification evaluation. Due to the well-established relationship between lysine and tryptophan content in maize protein (approximately 4:1), tryptophan can be used as a single parameter for evaluating nutritional quality of the protein [33, 34]. When interpreting results of laboratory analysis, tryptophan content and quality index have to be above the acceptable limits [8]. High level of tryptophan content was maintained throughout the protein quality improvement of ZPL 5. While protein content was significantly decreased (except for NIL 8 and NIL 9 in 2015), tryptophan content and quality index were increased in the 13 NILs by 37–50% and 42–56%, respectively. Different levels of tryptophan increase were achieved by MAS in some other researches. In two improved versions of parental lines obtained by Gupta et al. [32] tryptophan content increase of approximately 20% and less than 5% was found, while biochemical analysis of the improved lines in Babu et al. [20] and Jompuk et al. [35] showed that tryptophan concentration in protein was enhanced more than twice as compared to the original recipient lines. Moreover, an increased level of tryptophan content maintained during the selection in our research confirmed high heritability of this trait, as well as efficient action of amino acids modifier genes.

The first published report highlighting the importance of hard vitreous endosperm or “modified” grains in reducing the negative pleiotropic effects of the *o2* mutation was published in 1969 [36]. Selection for hard endosperm modification was rapidly incorporated into *o2* breeding schemes [8]. Small percentage of soft endosperm kernels of the analysed sub-lines in our research indicated that sufficient degree of endosperm modification was accomplished. Therefore, it could be assumed that undesirable characteristics such as kernel cracking and susceptibility to ear rots and pests are not to be expected and consequently higher grain yields could be achieved with the improved NILs.

During the conversion of ZPL 5 inbred line into quality protein NIL counterparts by MAS, several impediments occurred with the insufficient number of recessive homozygotes (*o2o2*) being the bottleneck in this process. Percentage of recessive homozygous individuals identified in BC_2_F_2_ generation of both crosses was lower (4.5% and 7.6%) than the expected 25% according to the Mendelian ratio of 1 *O2O2*: 2 *O2o2*: 1 *o2o2*. A small percentage of recessive homozygotes was also reported in Jompuk et al. [34]. They identified 8 out of 70 plants (8.57%) from Agron20 × Pop65C_6_-46 cross and only one out of 70 plants (1.43%) from Agron29 × Pop65C_6_-55 cross. As probable causes, these authors suggested either the small sample size or a random selection of plants from the field.

However, it is possible that number of *o2o2* plants can be affected by higher competitive ability of *O2* allele-bearing pollen. Sari-Gorla and Rovida [37] studied fertilization ability, the main component of the pollen competitive ability, using pollen mixture technique in which *opaque2* gene was used as a genetic marker in order to reveal the genetic sources of the two competing pollen types. Their research showed differences regarding germination rate of dominant and recessive allele-bearing pollen, in favour of the dominant type.

It was also shown that incompatible pollen grains are arrested at certain points in their journey toward the ovule [38]. The complex physiological relationships between pollen and style involve processes of stylar activation by the pollen tube, and activation of the pollen genome as an answer to stylar protein production. Differences in pollen competitive abilities could be due to differences in ability to induce metabolic responses in the style or to utilize products released by the style. A pilot study was conducted on *in vitro* pollen germination and *in vivo* pollen tube growth in parental line ZPL 5 and *O2o2* plants obtained in BC_2_F_2_ (unpublished data). The results proved pollen viability and penetration of pollen tubes in the proximal part of the silk and ovary within 24 hours after pollination in almost all genotypes. However, it was also shown that more than 50% of pollen grains were arrested at certain points, not reaching the ovule. These results point out to the incompatibility between pollen and style and could be the cause of another drawback in converting ZPL 5 to its high tryptophan counterparts—poor seed set throughout MAS. The insufficient kernel number was a limiting factor for selection and the reason for the loss of the ZPL 3 progenies, as well as many individual plants in different generations of selection.

In BC_2_ generation of ZPL 3 × CML 144 cross, three selected *o2o2* plants had higher genetic similarities with the recurrent parent than the two selected plants from BC_2_ generation of ZPL 5 × CML 144. This could be due to the larger BC_2_ population (329 versus 250 plants, respectively). Thus, it could be surmised that enlargement of a BC_2_ population increases the probability of reducing the time necessary for MAS, but it should not be accomplished at the expenses of higher investments. In any case, *o2o2* plants in the following generation of ZPL 3 conversion were lost and lack of recessive homozygotes was most probably the consequence of pollen incompatibility or low adaptability of *o2* pollen grains. Even at the last stage of selection in 2015, seven NILs originating from ZPL 5 were lost and another six had moderate seed set, while original ZPL 5 line had an excellent seed set. This year was characterized by severe drought, with extremely high temperatures and with no rainfalls in the period of five weeks, including the time of pollination [39]. Good seed set of *O2O2* ZPL 5 plants and incomplete seed set in *o2o2* NILs point out to the better adaptation of *O2* pollen to drought conditions.

The problems met during MAS would possibly have been avoided if exotic germplasm had not been used as a donor of *o2*. QPM was first developed for tropical and sub-tropical regions aimed for human consumption and these genotypes have been used as sources of essential amino acids for improving protein quality of maize genotypes adapted to temperate climate. The origins of ZPL 5 and CML 144 are quite distinct (temperate and tropical germplasm), what was confirmed by a very low GS coefficient value of 0.05 [25]. Low GS values are disadvantageous for backcross selection and the recovery of the recurrent parent's genome is harder to accomplish [40, 41]. Two BC and three generations of selfing provided 93% recovery of ZPL 5 genome and at least one more backcross generation should be done to achieve higher percentage of this recovery.

Influence of the exotic germplasm was also evident on the phenotypes of derived sub-lines. Significant differences were found for plant height and kernel row number between ZPL 5 and all analyzed sub-lines, as well as for some other traits in one to few sub-lines. Similar results were obtained in phenotypic analysis of test crosses. The majority of the analysed agronomic traits showed no significant differences between the hybrid created with ZPL 5 and hybrids with selected BC_2_F_3_ progenies, except for plant height, kernel row number, grain moisture and leaf number above the uppermost ear. Higher plants and higher number of kernel rows are characteristics of tropical and sub-tropical germplasm compared to temperate germplasm [42, 43], and all the sub-lines had significantly higher values for both traits than ZPL 5. Considering hybrids, plants were higher or at the level of the standard hybrid, while kernel row number was higher in all hybrids compared to the standard hybrid. Due to the displayed stability of these traits, it can be assumed that their values are the result of the tropical origin of the parental line CML 144.

The main setback to commercial use of QPM is that it frequently has low yields relative to non-QPM hybrids [18]. Different results for grain yield were achieved in different researches considering inbred lines and hybrids improved in protein quality by MAS. Superiority of the improved hybrids over their standard versions were obtained by Babu et al. [20] and Gupta et al. [31], who reported 9–12% and 3.5–13.8% increase in grain yield, respectively. On the other hand, Jompuk et al. [34] reported 7–75% decrease in grain yield of the experimental hybrids compared to commercial single cross hybrids. Across the nine QPM lines developed by Worral et al. [44], there was an 11.5% drop in grain yield compared to the commercial checks. However, in the same research the top-yielding QPM hybrids for each tester had similar yields to hybrids derived from commercial stock crossed with the same testers. In our research, sub-lines showed an increase in grain yield of 11–31% compared to ZPL 5, although this increase was not statistically significant. The only exception was regarding SL-4/1, which had significant increase in grain yield that could be explained as the consequence of gene dragging from tropical source of *o2* allele. Also, three out of four hybrids with selected BC_2_F_3_ families had higher yields than the hybrid created with parental line ZPL 5, while the remaining one had grain yield at the level of the standard hybrid with ZPL 5. These results point out that combining ability of the improved lines is on a par with the original ZPL 5 line.

In order to determine phenotypic traits that could be selected for during development of high tryptophan genotypes, correlation analysis between the estimated traits was performed. Positive but non-significant correlations were found between grain yield and biochemical parameters, which is partly in accordance with results reported in Zaidi et al. [45]. These authors found significant positive correlations between grain yield and both protein and tryptophan contents, but non-significant correlations between grain yield and quality index. Reddy et al. [46] also observed positive correlations between grain yield and tryptophan content as well as quality index, but negative correlation between grain yield and protein content. Negative correlations between grain yield and protein content were also found in several different researches [47–49], while Prakash et al. [50] found a positive correlation. Positive correlations between grain yield and biochemical traits found in our research indicate the possibility of simultaneous selection and improvement of these traits. Based on the correlations found between biochemical and phenotypic parameters, selection for plant height, ear length and kernel row number could be recommended together with tryptophan content for development of high quality protein maize genotypes with this material.

Finally, after two generations of backcrossing and three generations of selfing, six NILs with high tryptophan content, deriving from three sub-lines, were developed. The six NILs showed decrease of average protein content, and increase of average tryptophan content and quality index relative to the recurrent parent. Also, differences between the years of the analysis were not noticeable, although water surplus was present in 2014 and severe drought occurred in 2015 [38]. The only exception was NIL-9, which had much higher tryptophan content under drought conditions, as well as higher protein content and quality index. Different data on the change of protein and tryptophan contents under drought stress can be found in the literature. A significant protein decrease was obtained by Ali et al. [51] and a significant increase was found by Zaidi et al. [44]. In the latter paper, a significant increase for tryptophan and lysine contents under drought was also recorded. Similar results were obtained in Ignjatovic-Micic et al. [52]. Nevertheless, the six NILs displayed high tryptophan content and quality index regardless of the year, with average increments compared to ZPL 5 of 30% and 36%, respectively.

## Conclusions

Using molecular breeding approach, six NILs adapted to temperate regions with high protein quality, hard endosperm, improved grain yield and good combining abilities were developed. Although this approach proved to be efficient, small number of recessive homozygotes (*o2o2*) and poor seed set throughout MAS could be considered as the major impediments in backcross breeding for QPM, most probably due to the incompatibility between pollen and style. These problems could be the consequence of the exotic origin of the donor germplasm, which were also evident in some phenotypic traits, mainly plant height and kernel row number. Our results indicate that when using exotic germplasm for conversion of temperate genotypes, at least three backcrosses should be done for adequate recovery of the recurrent parent genome. Also, it could be assumed from our experiment that larger BC_2_ population would provide a sufficient number of *o2o2* plants with high percentage of recurrent parent genome and thus increase MAS efficiency. NILs created in this research are being used as *o2* donors in a marker assisted breeding program to convert several MRI commercial inbred lines to their high protein quality counterparts. It could be expected that the use of NILs as donors adapted to temperate climate and bearing high percentage of domestic germplasm will outbalance the impediments met in this research. Also, developed NILs can be considered the candidate parents for developing QPM hybrids adapted to temperate regions.

## Supporting Information

S1 Table List of SSR markers used in genetic similarity analysis. (DOCX)

S2 Table Phenotypic analysis of the recurrent parent ZPL 5 and the selected sub-lines. (DOCX)

S3 Table Phenotypic analysis of hybrids created with ZPL 5 (H-1) and with selected BC_2_F_3_ families (hybrids H-2 to H-5). (DOCX)
