# Supplementary material for: Development of High Tryptophan Maize Near Isogenic Lines Adapted to Temperate Regions through Marker Assisted Selection - Impediments and Benefits
Source: PLoS One. 2016 Dec 9;11(12):e0167635. doi: 10.1371/journal.pone.0167635 (PMC5147942; doi:10.1371/journal.pone.0167635)
Supplement: S1 Table — (DOCX) [file pone.0167635.s001.docx]

S1 Table List of SSR markers used in genetic similarity analysis.

|  | Name | Bin | Repeat | | Sequence (5’-3’)  (forward and reverse) |
| --- | --- | --- | --- | --- | --- |
|  |  |  |  |  |  |
| 1 | umc 1282 | 1.01 | (AT)6 | TACACTACACGACTCCCAACAGGA  GCGAGGGTTCTTTCCATAGAGAAT | |
| 2 | umc 1070 | 1.02 | (TC)7 | TTCCAGTAAGGGAGGTGCTG  TAAGCAACATATAGCCGGGC | |
| 3 | umc 1076 | 1.05 | CA | TTGGAAATCACCAATTGATATAGTTTG  TCTATTGCAAACGCCAAAAGTAGC | |
| 4 | umc 1335 | 1.06 | (AG)24 | ATGGCATGCATGTGTTTGTTTTAC  ACAGACGTCGCTAATTCCTGAAAG | |
| 5 | umc 1013 | 1.08 | (GA)9 | TAATGTGTCCATACGGTGGTGG  AGCTGGCTAGTCTCAGGCACTC | |
| 6 | bnlg 1643 | 1.08 | (AG)24 | ACCACCGTCCACCTCCAC  ATTGACCCCGTGACCCTC | |
| 7 | umc 2047 | 1.09 | (GACT)4 | GACAGACATTCCTCGCTACCTGAT  CTGCTAGCTACCAAACATTCCGAT | |
| 8 | umc 1605 | 1.12 | (GGC)4 | GGAGAAGCACGCCTTCGTATAG  CCAGGAGAGAAATCAACAAAGCAT | |
| 9 | umc 1265 | 2.02 | (TCAC)4 | GCCTAGTCGCCTACCCTACCAAT  TGTGTTCTTGATTGGGTGAGACAT | |
| 10 | bnlg 2248 | 2.03 | (AG)30 | CCACCACATCCGTTACATCA  ACTTTGACACCGGCGAATAC | |
| 11 | bnlg 1633 | 2.07 | (AG)16 | GTACCTCCAGGTTTACGCCA  TCAACTTCTCATGCACCCAT | |
| 12 | bnlg198 | 2.08 | - | GTTTGGTCTTGCTGAAAAATAAAA  GCTGGAGGCCTACATTATTATCTC | |
| 13 | bnlg 1520 | 2.09 | (AG)22 | TCCTCTTGCTCTCCATGTCC  ACAGCTGCGTAGCTTCTTCC | |
| 14 | phi 036 | 3.04 | AG | CCGTGGAGAGACGTTTGACGT  TCCATCACCACTCAGAATGTCAGTGA | |
| 15 | bnlg 197 | 3.06 | - | GCGAGAAGAAAGCGAGCAGA  CGCCAAGAAGAAACACATCACA | |
| 16 | bnlg 1350 | 3.08 | (AG)13 | TGCTTCAGCGCATTAAACTG  TGCTCGTGTGAGTTCCTACG | |
| 17 | umc 1594 | 3.09 | (TA)10 | CACTGCAGGCCACACATACATA  GCCAGGGGAGAAATAAAATAAAGC | |
| 18 | phi 072 | 4.00 | AAAC | ACCGTGCATGATTAATTTCTCCAGCCTT  GACAGCGCGCAAATGGATTGAACT | |
| 19 | umc 2039 | 4.03 | (CAG)5 | CATCTCCTACCAGCTCACCCC  GCTCGGGGTAGTAGTGTTCTCCTT | |
| 20 | umc 1418 | 4.08 | (GGAAG)4 | TCACACACACACTACACTCGCAAT  GAGCCAAGAGCCAGAGCAAAG | |
| 21 | bnlg 589 | 4.10 | - | GGGTCGTTTAGGGAGGCACCTTTGGT  GCGACAGACAGACAGACAAGCGCATTGT | |
| 22 | umc 1109 | 4.10 | (ACG)4 | TCACACACACACTACACTCGCAAT  GAGCCAAGAGCCAGAGCAAAG | |
| 23 | bnlg 557 | 5.03 | - | TCACGGGCGTAGAGAGAGA  CGAAGAAACAGCAGGAGATGAC | |
| 24 | umc 1274 | 5.03 | (TGC)5 | TTGAGTCTGGTACTGCGTATGAGG  TAGCACTCCAACAGCAAGAGTTTG | |
| 25 | phi 085 | 5.06 | AACGC | AGCAGAACGGCAAGGGCTACT  TTTGGCACACCACGACGA | |
| 26 | phi 087 | 5.06 | ACC | GAGAGGAGGTGTTGTTTGACACAC  ACAACCGGACAAGTCAGCAGATTG | |

S1 Table Continued.

|  | Name | | Bin | | Repeat | | Sequence  (*forward* i *reverse*) |  |
| --- | --- | --- | --- | --- | --- | --- | --- | --- |
|  |  |  |  |  |  |  |  |  |
| 27 | phi 075 | 6.00 | | CT | | GGAGGAGCTCACCGGCGCATAA  AAAGGTTACTGGACAAATATGCGTAACTCA | | |
| 28 | umc 1006 | 6.02 | | (GA)19 | | AATCGCTTACTTGTAACCCACTTG  AGTTTCCGAGCTGCTTTCTCT | | |
| 29 | mmc 0241 | 6.05 | | (TA)4N13  (TG)15 | | TATATCCGTGCATTTACGTTT  CATCGCTTGTCTGTCGA | | |
| 30 | bnlg 1443 | 6.05 | | (AG)25 | | TACCGGAATCCTCTTTGGTG  TTTGACAACCTCTTCCAGGG | | |
| 31 | umc 1695 | 7.00 | | (CA)8 | | CAGGTAATAACGACGCAGCAGAA  GTCCTAGGTTACATGCGTTGCTCT | | |
| 32 | umc 1036 | 7.02 | | GA | | CTGCTGCTCAAGGAGATGGAGA  GACACACATGCACGAGCAGACT | | |
| 33 | umc 1393 | 7.02 | | (GTC)4 | | CCTTCTTCTTATTGTCACCGAACG  GCCGATGAGATCTTTAACAACCTG | | |
| 34 | umc 1015 | 7.03 | | (GA)45 | | CAGACACAAGCAGCAAAGCAAG  TCCGACTCCAAGAAGAGGAGAA | | |
| 35 | umc 1324 | 7.03 | | (AGC)5 | | ATCCATCATCATCATCATTGCTTG  ATGTCATCATGTACCAGGTGTTGG | | |
| 36 | umc 1782 | 7.04 | | (GAC)4 | | CGTCAACTACCTGGCGAAGAA  TCGCATACCATGATCACTAGCTTC | | |
| 37 | umc 1799 | 7.04 | | (TG)12 | | GTGATGAATAATGTCCCCAATTCC  GGACAGATGTCTGGAGATTGCTTT | | |
| 38 | phi 116 | 7.06 | | | ACTG/ACG | GCATACGGCCATGGATGGGA  TCCCTGCCGGGACTCCTG | | |
| 39 | bnlg 2235 | 8.02 | | | (AG)23 | ATCCGGAGACACATTCTTGG  CTGCAAGCAACTCTCATCGA | | |
| 40 | umc 1858 | 8.04 | | | (TA)8 | GTTGTTCTCCTTGCTGACCAGTTT  ATCAGCAAATTAAAGCAAAGGCAG | | |
| 41 | phi 080 | 8.08 | | | AGGAG | CACCCGATGCAACTTGCGTAGA  TCGTCACGTTCCACGACATCAC | | |
| 42 | phi 033 | 9.01 | | | AAG | ATCGAAATGCAGGCGATGGTTCTC  ATCGAGATGTTCTACGCCCTGAAGT | | |
| 43 | umc 1040 | 9.01 | | | (CT)11 | CATTCACTCTCTTGCCAACTTGA  AGTAAGAGTGGGATATTCTGGGAGTT | | |
| 44 | bnlg 127 | 9.03 | | | - | CATGTATACGAGAAGCACCCTAT  ATCGTAACTCAGCGGTTTGTG | | |
| 45 | umc 1492 | 9.04 | | | (GCT)4 | GAGACCCAACCAAAACTAATAATCTCTT  CTGCTGCAGACCATTTGAAATAAC | | |
| 46 | umc 1771 | 9.04 | | | (CGTC)4 | GTGAAATGTTGTTTCCAATGCAAG  CATCAGGAAGGAAGACGACTAGGA | | |
| 47 | umc 1507 | 10.04 | | | (CACAA)4 | GATTCAAACCAAACACTTTTCCCA  CGAACCTTGCTGTGTGTTTATCAG | | |
| 48 | bnlg 1526 | 10.04 | | | (AG)15 | ACGAGCGAGTGGAGAATAGG  AGCCCAGTACGTGGGGTC | | |
| 49 | umc 1506 | 10.05 | | | (AACA)4 | AAAAGAAACATGTTCAGTCGAGCG  ATAAAGGTTGGCAAAACGTAGCCT | | |
| 50 | umc 1827 | 10.05 | | | (GAC)6 | GCAAGTCAGGGAGTCCAAGAGAG  CCACCTCACAGGTGTTCTACGAC | | |

- data not available
