# Supplementary material for: Development of High Tryptophan Maize Near Isogenic Lines Adapted to Temperate Regions through Marker Assisted Selection - Impediments and Benefits
Source: PLoS One. 2016 Dec 9;11(12):e0167635. doi: 10.1371/journal.pone.0167635 (PMC5147942; doi:10.1371/journal.pone.0167635)
Supplement: S2 Table — (DOCX) [file pone.0167635.s002.docx]

S2 Table Phenotypic analysis of the recurrent parent ZPL 5 and the selected sub-lines.

| Genotype | ASI^a^ | PH^b^  (cm) | EH^c^  (cm) | LN^d^ | LNE^e^ | ENP^f^ | BP^g^  (%) | CP^h^  (%) | GM^i^  (%) | EL^j^  (cm) | KRN^k^ | NKR^l^ | KL^m^  (cm) | HKW^n^  (g) | GY^o^  (t/ha) |
| --- | --- | --- | --- | --- | --- | --- | --- | --- | --- | --- | --- | --- | --- | --- | --- |
| ZPL 5 | 3.00a | 168.90d | 47.25b | 13.43de | 4.10d | 0.81b | 6.72ab | 18.62ab | 17.25b | 14.94cd | 12.75c | 30.59bc | 0.77bc | 18.05b | 3.16bc |
| SL-4/1 | 3.25a | 217.30a | 73.03a | 16.10a | 5.50a | 1.02a | 3.12b | 19.40ab | 19.94a | 18.69a | 15.85ab | 39.01a | 1.00a | 25.54a | 8.59a |
| SL-5/1 | 4.25a | 185.40c | 50.03b | 13.65cde | 4.38cd | 0.80b | 12.67a | 21.38a | 17.10b | 14.60d | 15.58ab | 27.95c | 0.73c | 18.05b | 2.46c |
| SL-5/2 | 2.50a | 187.38c | 45.60b | 13.23de | 4.55bcd | 0.80b | 3.49b | 16.38b | 17.44b | 16.86b | 15.50ab | 34.90ab | 0.77bc | 15.67b | 3.77bc |
| SL-5/3 | 4.25a | 195.63bc | 51.88b | 13.93bcd | 4.75bc | 0.78b | 10.10ab | 18.30ab | 17.19b | 16.36bc | 15.60ab | 32.79bc | 0.80bc | 16.92b | 3.50bc |
| SL-5/4 | 3.50a | 186.08c | 47.00b | 13.23de | 4.58bcd | 0.88ab | 3.12b | 17.42b | 17.49b | 17.08ab | 15.45ab | 35.61ab | 0.77bc | 15.37b | 4.14bc |
| SL-6/2 | 3.75a | 202.68b | 54.93b | 14.45b | 4.88b | 0.90ab | 9.81ab | 18.45ab | 17.06b | 16.19bcd | 16.85a | 32.85bc | 0.85b | 16.58b | 4.26b |
| SL-6/3 | 4.00a | 197.95bc | 54.25b | 14.25bc | 4.65bc | 0.90ab | 3.35b | 19.25ab | 16.81b | 16.90b | 15.10b | 32.88bc | 0.76bc | 17.55b | 3.50bc |
| SL-6/4 | 3.00a | 189.48c | 46.18b | 13.10e | 4.28cd | 0.94ab | 13.39a | 16.65b | 16.84b | 17.19ab | 15.45ab | 32.89bc | 0.78bc | 17.66b | 3.88bc |
| Mean | 3.50 | 192.31 | 52.24 | 13.93 | 4.63 | 0.87 | 7.31 | 18.43 | 17.46 | 16.53 | 15.34 | 33.27 | 0.80 | 17.93 | 4.14 |
| SD^p^ | 1.65 | 15.44 | 11.23 | 1.09 | 0.50 | 0.13 | 6.22 | 2.48 | 1.10 | 1.66 | 1.36 | 4.05 | 0.10 | 3.31 | 2.04 |
| CV^q^(%) | 39.27 | 4.00 | 13.13 | 3.55 | 6.58 | 14.19 | 65.60 | 11.83 | 3.50 | 6.32 | 6.49 | 9.42 | 8.09 | 11.63 | 25.07 |
| LSD_0.05_^r^ | 2.24 | 12.55 | 11.19 | 0.81 | 0.50 | 0.20 | 7.82 | 3.56 | 1.00 | 1.70 | 1.62 | 5.11 | 0.10 | 3.40 | 1.69 |

^a^ASI - anthesis-silking interval, ^b^PH - plant height, ^c^EH - ear height, ^d^LN - leaf number, ^e^LNE - leaf number above the uppermost ear, ^f^ENP - ear number per plant, ^g^BP - percentage of broken plants, ^h^CP - cob percentage, ^i^GM - grain moisture, ^j^EL - ear length, ^k^KRN - kernel row number, ^l^NKR - number of kernels per row, ^m^KL - kernel length, ^n^HKW - hundred kernel weight, ^o^GY - grain yield, ^p^SD - standard deviation, ^q^CV - coefficient of variation, ^r^LSD_0.05_ - least significant difference at 0.05 level. Means followed by the same letter(s) within the same columns are not significantly different at 0.05 level
