# Supplementary material for: Development of High Tryptophan Maize Near Isogenic Lines Adapted to Temperate Regions through Marker Assisted Selection - Impediments and Benefits
Source: PLoS One. 2016 Dec 9;11(12):e0167635. doi: 10.1371/journal.pone.0167635 (PMC5147942; doi:10.1371/journal.pone.0167635)
Supplement: S3 Table — (DOCX) [file pone.0167635.s003.docx]

S3 Table Phenotypic analysis of hybrids created with ZPL 5 (H-1) and with selected BC_2_F_3_ families (hybrids H-2 to H-5).

| Genotype | ASI^a^ | PH^b^  (cm) | EH^c^  (cm) | LN^d^ | LNE^e^ | ENP^f^ | BP^g^  (%) | CP^h^  (%) | GM^i^  (%) | EL^j^  (cm) | KRN^k^ | NKR^l^ | KL^m^  (cm) | HKW^n^  (g) | GY^o^  (t/ha) |
| --- | --- | --- | --- | --- | --- | --- | --- | --- | --- | --- | --- | --- | --- | --- | --- |
| H-1 | 2.00a | 220.18b | 84.18a | 14.52a | 4.02b | 1.00a | 7.81a | 14.15a | 18.42b | 17.99ab | 13.10b | 36.24a | 1.12a | 37.20a | 10.07ab |
| H-2 | 2.25a | 238.85a | 90.98a | 15.30a | 4.48ab | 1.00a | 4.69a | 14.28a | 18.62b | 19.02a | 14.15a | 36.22a | 1.16a | 36.51a | 11.93a |
| H-3 | 2.50a | 228.22b | 84.92a | 15.18a | 4.58a | 0.95a | 3.12a | 15.33a | 19.56a | 18.32ab | 14.25a | 36.50a | 1.11a | 36.88a | 11.64ab |
| H-4 | 2.00a | 236.42a | 85.78a | 15.25a | 4.60a | 0.90a | 1.56a | 14.08a | 19.24ab | 18.42ab | 13.80a | 37.28a | 1.15a | 36.82a | 10.15ab |
| H-5 | 1.70a | 220.20b | 84.70a | 14.98a | 4.45ab | 0.90a | 6.25a | 15.22a | 18.42b | 17.00b | 13.75a | 33.06a | 1.09a | 36.74a | 9.67b |
| Mean | 2.1 | 228.78 | 86.11 | 15.04 | 4.42 | 0.95 | 4.69 | 14.62 | 18.86 | 18.15 | 13.81 | 35.86 | 1.13 | 36.83 | 10.69 |
| SD^p^ | 0.64 | 14.51 | 6.89 | 0.50 | 0.36 | 0.07 | 6.37 | 1.40 | 0.74 | 1.37 | 0.68 | 4.47 | 0.07 | 1.19 | 2.41 |
| CV^q^(%) | 30.12 | 1.81 | 4.12 | 3.62 | 5.87 | 8.15 | 142.98 | 9.00 | 2.52 | 4.86 | 2.07 | 6.35 | 6.86 | 4.15 | 10.30 |
| LSD_0.05_^r^ | 1.24 | 8.13 | 9.84 | 1.51 | 0.51 | 0.15 | 13.16 | 2.58 | 0.94 | 1.73 | 0.56 | 4.47 | 0.15 | 3.00 | 2.16 |

^a^ASI - anthesis-silking interval, ^b^PH - plant height, ^c^EH - ear height, ^d^LN - leaf number, ^e^LNE - leaf number above the uppermost ear, ^f^ENP - ear number per plant, ^g^BP - percentage of broken plants, ^h^CP - cob percentage, ^i^GM - grain moisture, ^j^EL - ear length, ^k^KRN - kernel row number, ^l^NKR - number of kernels per row, ^m^KL - kernel length, ^n^HKW - hundred kernel weight, ^o^GY - grain yield, ^p^SD - standard deviation, ^q^CV - coefficient of variation, ^r^LSD_0.05_ - least significant difference at 0.05 level. Means followed by the same letter(s) within the same columns are not significantly different at 0.05 level
